# Early Outcomes of Transcatheter Aortic Valve Implantation for Severe Aortic Stenosis With Myval Versus Edwards Sapien 3 Valve: A Systematic Review and Meta-Analysis

**DOI:** 10.7759/cureus.96410

**Published:** 2025-11-09

**Authors:** Dinkar Bhasin, Ankita Bhasin, Tsering Sangdup, Arun Sharma, Prashant Panda, Yash Paul Sharma

**Affiliations:** 1 Cardiology, Postgraduate Institute of Medical Education and Research, Chandigarh, IND; 2 Anesthesiology, Government Medical College & Hospital, Chandigarh, IND; 3 Cardiothoracic Surgery, Postgraduate Institute of Medical Education and Research, Chandigarh, IND; 4 Radiology, Postgraduate Institute of Medical Education and Research, Chandigarh, IND

**Keywords:** aortic stenosis, meta-analysis, myval, sapien, systematic review, tavi, tavr, thv

## Abstract

The Edwards Sapien (Edwards Lifesciences, Irvine, CA, USA) transcatheter heart valves (THVs) and the Myval THVs (Meril Life Sciences, Vapi, Gujarat, India) are two balloon-expandable valve systems used for transcatheter aortic valve implantation (TAVI). This systematic review and meta-analysis aimed to compare the clinical and hemodynamic outcomes at 30 days after TAVI using the Myval versus the Edwards Sapien 3 valve in patients with severe aortic stenosis (AS). We conducted a systematic search of PubMed, Embase, Cochrane Library, and clinicaltrials.gov up to June 21, 2025. The inclusion criteria were observational studies or interventional trials comparing Myval or Myval Octacor with Edwards Sapien 3 or Sapien 3 Ultra in patients with severe AS undergoing TAVI. The risk of bias was assessed using the Cochrane Risk of Bias Tool 2 for randomized controlled trials (RCTs) and the Newcastle-Ottawa Scale for observational studies. Pooled estimates were derived using a random-effects model. The primary outcomes were all-cause mortality, new permanent pacemaker implantation (PPI), and major vascular complications assessed at 30 days. A predefined subgroup analysis was performed based on the study design. The study protocol was registered on the INPLASY database (INPLASY202560110). Four studies (two retrospective cohort and two RCTs) with 1973 patients (18.6% in observational and 81.4% in RCTs) met the inclusion criteria. Out of the total, 1081 (54.8%) patients received Myval and 892 (45.2%) received Sapien 3 valves. No significant difference was observed in all-cause mortality (risk ratio (RR): 1.14, 95% CI: 0.55-2.37; p = 0.73; I^2 ^= 0), rate of new PPI (RR: 0.92, 95% CI: 0.49-1.74; p = 0.81; I^2 ^= 82%), or major vascular complications (RR: 0.70, 95% CI: 0.19-2.52; p = 0.58; I^2 ^= 33%) at 30 days. The findings were consistent in the subgroup analysis among observational studies and RCTs. Moderate to severe aortic regurgitation (AR) (RR: 2.58, 95% CI: 1.14-5.84; p = 0.02; I² = 0%) and new-onset atrial fibrillation (AF) (RR: 2.0, 95% CI: 1.10-3.63; p = 0.02; I² = 0%) were higher with Myval. Mean gradient was lower (mean difference: -2.85 mmHg, 95% CI: -3.88 to -1.82 mmHg; p < 0.001; I² = 82%) and effective orifice area was larger (mean difference: 0.28 cm^2^, 95% CI: 0.16-0.40 cm^2^; p < 0.001; I² = 84%) with Myval. Other secondary outcomes and procedural outcomes were comparable. To conclude, primary outcomes were comparable between the two THVs. The Myval had better hemodynamic parameters at 30 days but a higher rate of moderate to severe AR and new-onset AF. Further studies are needed to assess medium- and long-term outcomes between the two valves.

## Introduction and background

Aortic stenosis (AS) is a progressive, degenerative disease of the aortic valve [1]. In elderly patients with symptomatic severe AS, transcatheter aortic valve implantation (TAVI) is the preferred treatment modality in those with suitable anatomy and acceptable procedural risk [2]. TAVI is less invasive than surgical aortic valve replacement and has demonstrated comparable clinical outcomes [3,4]. Various transcatheter heart valves (THVs) have been developed and approved for TAVI, being broadly categorized into balloon-expandable and self-expanding valves.

Among balloon-expandable valves, Edwards Sapien valve series (Edwards Lifesciences, Irvine, CA, USA) is widely used with extensive clinical data [5,6]. Myval THV (Meril Life Sciences, Vapi, Gujarat, India) is a newer balloon-expandable valve that received its CE-mark in 2019 [7]. It has gained attention due to its catheter design with the valve pre-mounted on the balloon and its availability in intermediate sizes [7].

Clinical endpoints for TAVI, as defined by the Valve Academic Research Consortium, provide a standardized framework for evaluating procedural success and clinical outcomes of the TAVI procedure [8]. Comparative evaluation of different THVs using these endpoints is crucial for guiding evidence-based valve selection in clinical practice. Multiple observational studies and at least two major randomized controlled trials have compared the newer Myval valve with contemporary THVs, including Sapien and Evolut [9-14]. 

The existing studies have limited sample sizes, were powered for composite outcomes, and employed varying endpoint definitions. Hence, we conducted a systematic review and meta-analysis to pool the available evidence comparing early clinical and hemodynamic outcomes in patients undergoing TAVI for severe AS with Myval versus Edwards Sapien 3 THVs.

## Review

Methods 

This study was conducted in line with the Preferred Reporting Items for Systematic Reviews and Meta-Analyses (PRISMA) 2020 guidelines [15]. The protocol was prospectively registered in the INPLASY database (INPLASY202560110).

Literature Search

The search was conducted on 21st June 2025, on PubMed, Embase, and Cochrane Central Register of Controlled Trials (CENTRAL) through the Cochrane Library. The trial register, clinicaltrials.gov, was also searched. The search comprised of the broad terms: ‘TAVI,’ “trans-catheter aortic valve implantation,” “transcatheter aortic valve replacement,” “TAVR,” “Myval,” and “Myval valve,” “Myval Octacor,” and “aortic stenosis.” The search string was generated by utilizing controlled vocabulary, such as MeSH and Emtree, free text keywords, and synonyms without any restrictions. The elaborative search strategy is presented in the Appendix (see Table 2).

Eligibility Criteria 

Eligible studies included either observational studies or interventional trials comparing Myval or Myval Octacor valve with the Edwards Sapien 3 valve series (Sapien 3 or Sapien 3 Ultra) in patients with severe AS undergoing TAVI. To be eligible, the study should have been published in peer-reviewed journal and evaluated at least one of the primary or secondary outcomes.

Outcomes 

The primary outcomes were assessed at 30 days and included all-cause mortality, new permanent pacemaker implantation (PPI), and major vascular complications. The secondary outcomes were technical success, device success, major bleeding, all strokes, minor vascular complications, significant acute kidney injury (AKI), moderate to severe aortic regurgitation (AR), new-onset atrial fibrillation (AF), and hemodynamic parameters, including mean gradient and effective orifice area (EOA), with all outcomes being assessed at 30 days. Outcomes were defined as per the Valve Academic Research Consortium (VARC) criteria [8]. In addition, as an exploratory analysis, we also compared the procedural outcomes of procedural death, annular rupture, coronary occlusion, valve malposition, and tamponade by pooling data from eligible studies. 

Study Selection and Data Extraction

The results yielded by the search strategy were collated and duplicates removed. Full texts of the shortlisted studies were retrieved. The deduplicated reports were independently screened using the title and abstract. Relevant data from the eligible reports were extracted in a predefined data entry form. This included study identifiers, study and baseline patient characteristics, procedural details, and outcome data of three primary, ten secondary, and procedural outcomes. All steps were independently performed by two reviewers (A.B. and D.B.). Discrepancies were resolved through discussion with a third reviewer (T.S.).

Risk of Bias Assessment

Assessment for risk of bias was done independently by two reviewers (A.B. and D.B.) using the Cochrane Risk of Bias Tool 2 (RoB 2) for randomized controlled trials (RCTs) and the Newcastle-Ottawa Scale (NOS) for observational studies [16,17]. Any disagreements were resolved through discussion with a third reviewer (T.S.). 

Data Synthesis and Analysis 

For dichotomous variables, effect estimates were calculated as risk ratios (RRs) with 95% confidence intervals (CI), and continuous data were represented using mean difference and standard deviation. Pooled estimates were derived using a random-effects model and summarized in the forest plot with 95% CI. Wherever the original study reported median with interquartile range, it was converted into mean ± standard deviation using recommended formulas [18,19]. Heterogeneity between the studies was analyzed using the Cochran's Q test and expressed as the I² statistic. The interpretation of I² values was done as recommended by the Cochrane Handbook [20]. If the value of I^2^ was between 0% and 40% heterogeneity may not be important; between 30% and 60% may suggest a moderate degree of heterogeneity; 50% and 90% suggest a presence of substantial heterogeneity; and 75% and 100% suggest a considerable degree of heterogeneity [20].

A predefined subgroup analysis was performed by grouping RCTs and observational studies separately. The analysis was performed where at least two studies were present in either subgroup. Sensitivity analysis was performed for the primary outcome using a leave-one-out approach [21].

The software and online tools used at various stages of the review included Rayyan, EndNote (Clarivate, Philadelphia, PA), and Review Manager from the Cochrane Collaboration [22]. Data synthesis was performed by using RevMan 5 (Review Manager (RevMan), (Computer program), Version 5.4, The Cochrane Collaboration, 2020). The RoB 2 plots were generated using the online robvis tool [23].

Results

Search Results

**Figure 1:**
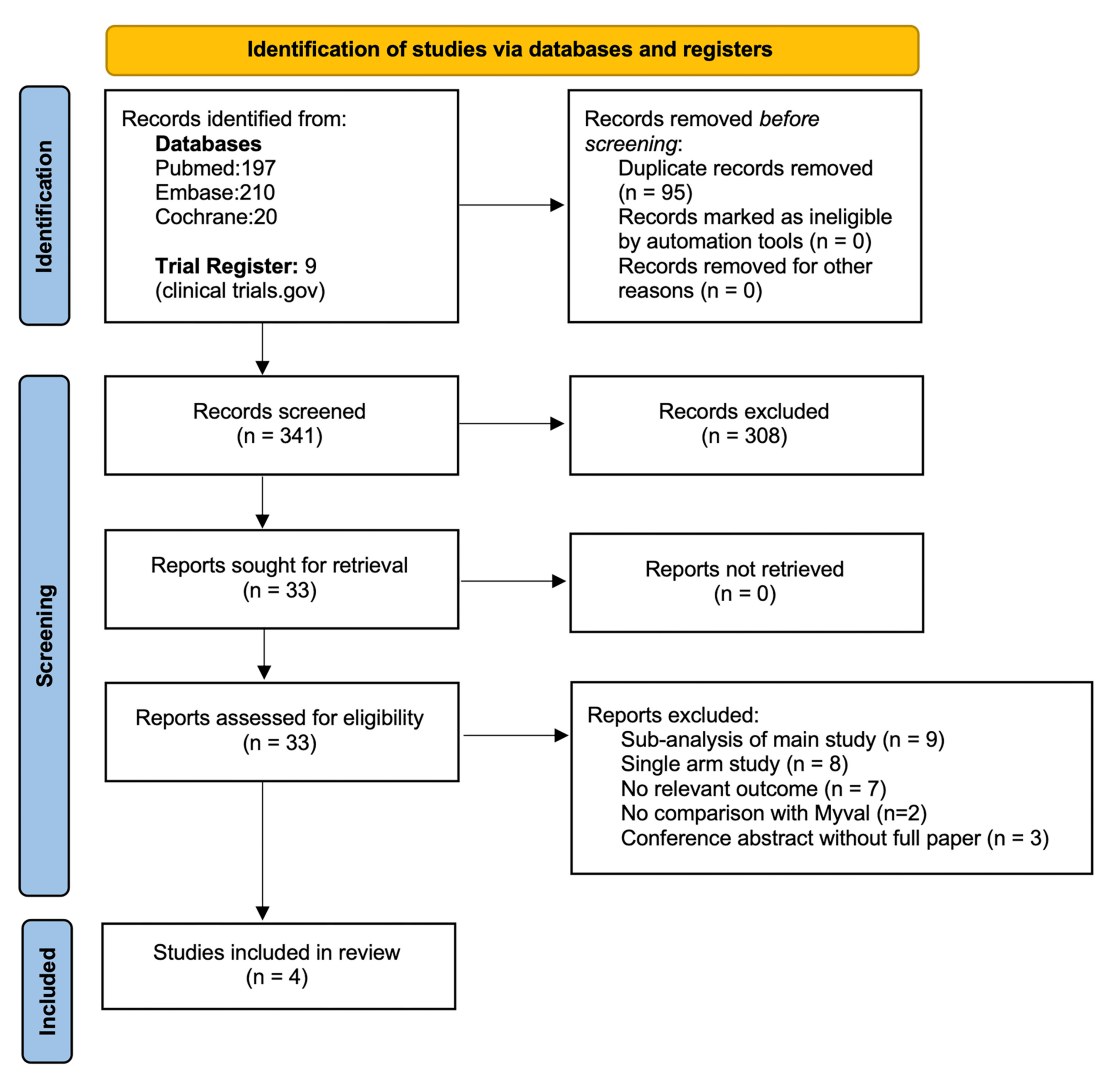
PRISMA flow diagram. PRISMA: Preferred Reporting Items for Systematic Reviews and Meta-Analyses.

The database search yielded 436 results. Ninety-five duplicates were removed. Titles and abstracts of 341 reports were screened against the eligibility criteria, and 308 reports were excluded. The full texts of 33 reports were reviewed, yielding four eligible studies for the systematic review [9,10,14,24]. The flow is summarized in the PRISMA flow diagram in Figure 1. Two other studies, Santos-Martinez et al. and Ubben et al., compared in-hospital outcomes between Myval and Sapien 3 but did not report any 30-day outcomes [11,12]. Hence, they were excluded from the systematic review. Details of excluded studies are provided in the Appendix (see Table 3).

Study and Baseline Patient Characteristics

A total of four studies, two RCTs and two retrospective cohort studies, involving 1973 patients, were included in the review [9,10,14,24]. Two RCTs contributed 1607 patients (81.4%) while the observational studies contributed 366 patients (18.6%). Of the entire patient pool, 1081 (54.8%) received Myval and 892 (45.2%) received Sapien 3. All studies were multicentric with predominant participation of European centers. All studies were published between 2021 and 2025.

Among the observational studies, Delgado-Arana et al. excluded bicuspid aortic valves, while the TRITON study only included patients with bicuspid aortic valves [9,10]. The RCTs, COMPARE-TAVI 1 trial, and the LANDMARK substudy comparing Sapien 3 and Myval as reported by van Royen et al., included patients with tricuspid and bicuspid aortic valves, with the proportion of the latter being <10% [14,24]. The COMPARE-TAVI 1 being an all-comers RCT also included patients undergoing valve-in-valve procedures, amounting to 4% of the population [14]. 

Three out of the four studies used the VARC-3 criteria for assessing outcomes, except for Delgado-Arana et al., which reported the outcomes according to the VARC-2 criteria [25]. Basic study characteristics are summarized in Table 1. Other details, including endpoints and inclusion criteria, are presented in the Appendix (see Table 4).

**Table 1:** Characteristics of included studies. VARC: Valve Academic Research Consortium.

Study authors and year	Year	Location	Centers	Study design	Intervention	Comparator	VARC criteria	Myval (n)	Sapien 3 (n)	Follow-up
Delgado-Arana et al. 2021 [9]	2021	Europe	Muticentric	Observational; matched retrospective cohort	Myval	Edwards Sapien 3	2	103	103	30 days
Amat-Santos et al. 2023 (TRITON) [10]	2023	Europe	Multicentric	Observational; matched retrospective registry-based cohort	Myval	Edwards Sapien 3	3	80	80	30 days
van Royen et al. 2025 (LANDMARK study) [24]	2025	Europe, Brazil, New Zealand	Multicentric	Randomized, open-label trial	Myval (91.3%) and Myval Octacor (8.7%)	Edwards Sapien 3 (55.4%) or Edwards Sapien 3 Ultra (44.6%)	3	384	192	30 days
Terkelsen et al. 2025 (COMPARE-TAVI 1) [14]	2025	Denmark	Multicentric	Randomized, open-label trial	Myval (36%) and Myval Octacor (63%)	Edwards Sapien 3 and Sapien 3 Ultra	3	514	517	30 days, one year

The average age of patients in all the studies was over 80 years, with the exception of the TRITON study, where the mean age was 77 years, possibly because it only recruited patients with bicuspid aortic valve who present at a younger age [10]. Over 40% of patients had a functional class of New York Heart Association (NYHA) class III or IV in three of the four studies that reported it. The mean Society of Thoracic Surgeons (STS) score was less than 4% in all four studies, suggesting an overall intermediate-risk population. Relevant baseline characteristics of the study patients, including clinical, echocardiographic, and computed tomography parameters, have been summarized in the Appendix (see Tables 5-7).

Procedural characteristics were similar across the studies except for some differences. Three of the four studies used only transfemoral access, except for van Royen et al., in which alternative access was used in <2% of patients [24]. Predilation was done more often in the Myval group, in almost 40% of the patients, except for the TRITON study, where it was done in over 70% patients [10]. Intermediate sizes were used in over 40% of the patients receiving Myval in individual studies, except for the TRITON study, where the figure was 25% [10]. The procedural characteristics across the studies are presented in the Appendix (see Table 8).

Risk of Bias Assessment

The RoB 2 tool was used at the outcome level. There were some concerns with regards to the risk of bias for both studies across all outcomes. The LANDMARK trial had some concerns due to deviation from the intended intervention, as 4% of patients in the Myval group received Sapien 3 [24]. The COMPARE-TAVI 1 study had some concerns in the selection of reported results, as there was no predefined protocol and statistical analysis plan for the study [14]. The RoB 2 assessment for various outcomes is presented in the Appendix (see Figure 7). Both the observational studies, Delgado-Arana et al. and the TRITON study were of high quality as assessed using the NOS. The NOS score for various domains is summarized in the Appendix (see Table 9). 

Quantitative Synthesis

**Figure 2:**
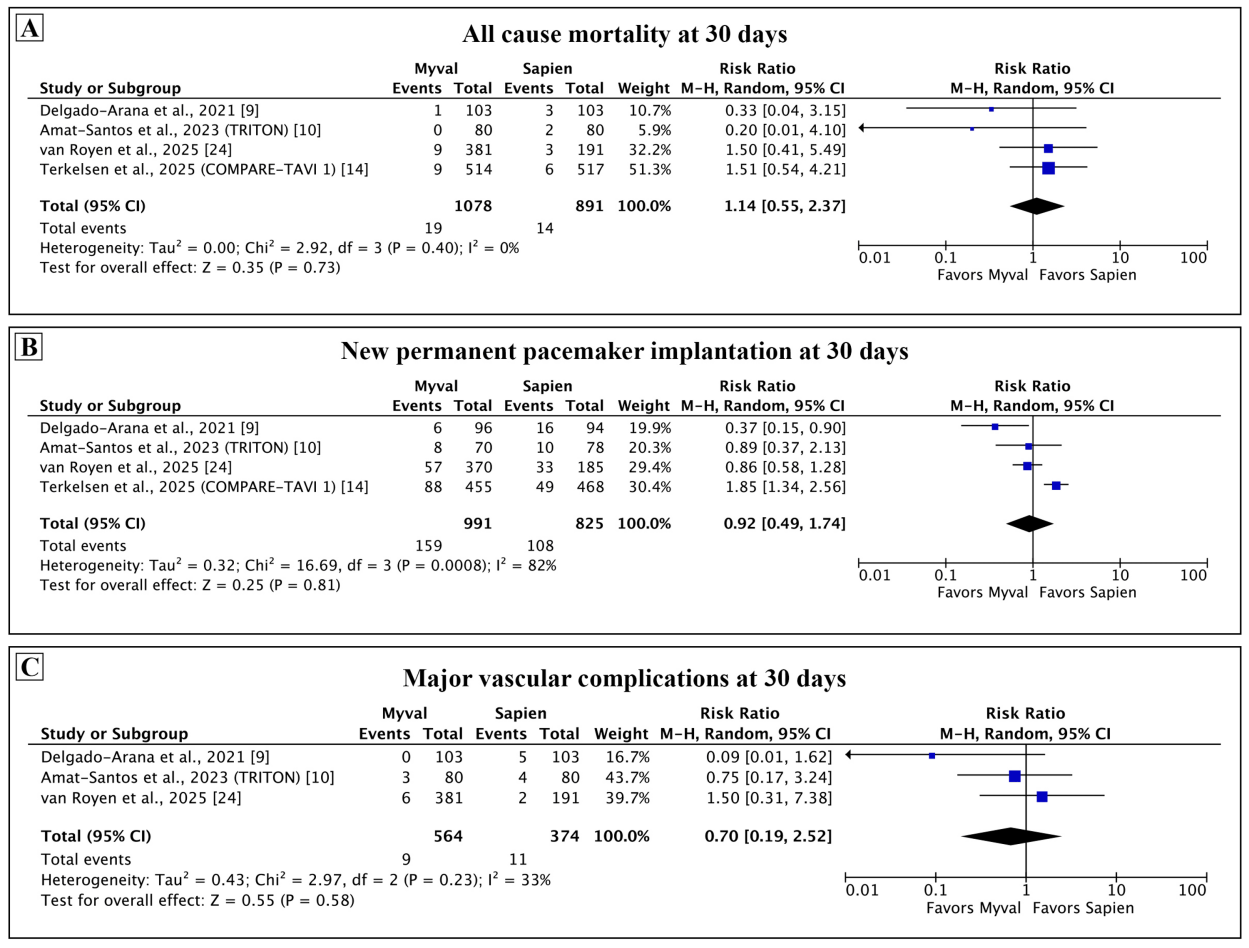
Comparison of primary outcomes between Myval and Sapien 3 at 30 days. Meta-analysis of the primary outcomes of all-cause mortality (A), new permanent pacemaker implantation (B), and major vascular complications (C).

Primary outcomes: The findings of the meta-analysis for the primary outcome at 30 days are presented in Figure 2. There was no statistical difference in the pooled estimate of all-cause mortality (RR: 1.14, 95% CI: 0.55-2.37; p = 0.73; I² = 0%). The finding was consistent across all studies, and heterogeneity for this comparison was low. Three out of four studies reported the rate of PPI as a proportion of all patients in each arm and did not exclude those with pre-existing PPI. We calculated the rate of new PPI in each study as a proportion of patients without pre-existing PPI that received PPI after TAVI. The pooled estimate of new PPI at 30 days was similar between the two groups (RR: 0.92, 95% CI: 0.49-1.74; p = 0.81; I² = 82%). Sensitivity analysis for this outcome was performed by using the originally reported rates of new PPI in the individual studies and showed similar findings (see Appendix, Figure 8). The rate of major vascular complications was comparable with low levels of heterogeneity (RR: 0.70, 95% CI: 0.19-2.52; p = 0.58; I² = 33%). 

**Figure 3:**
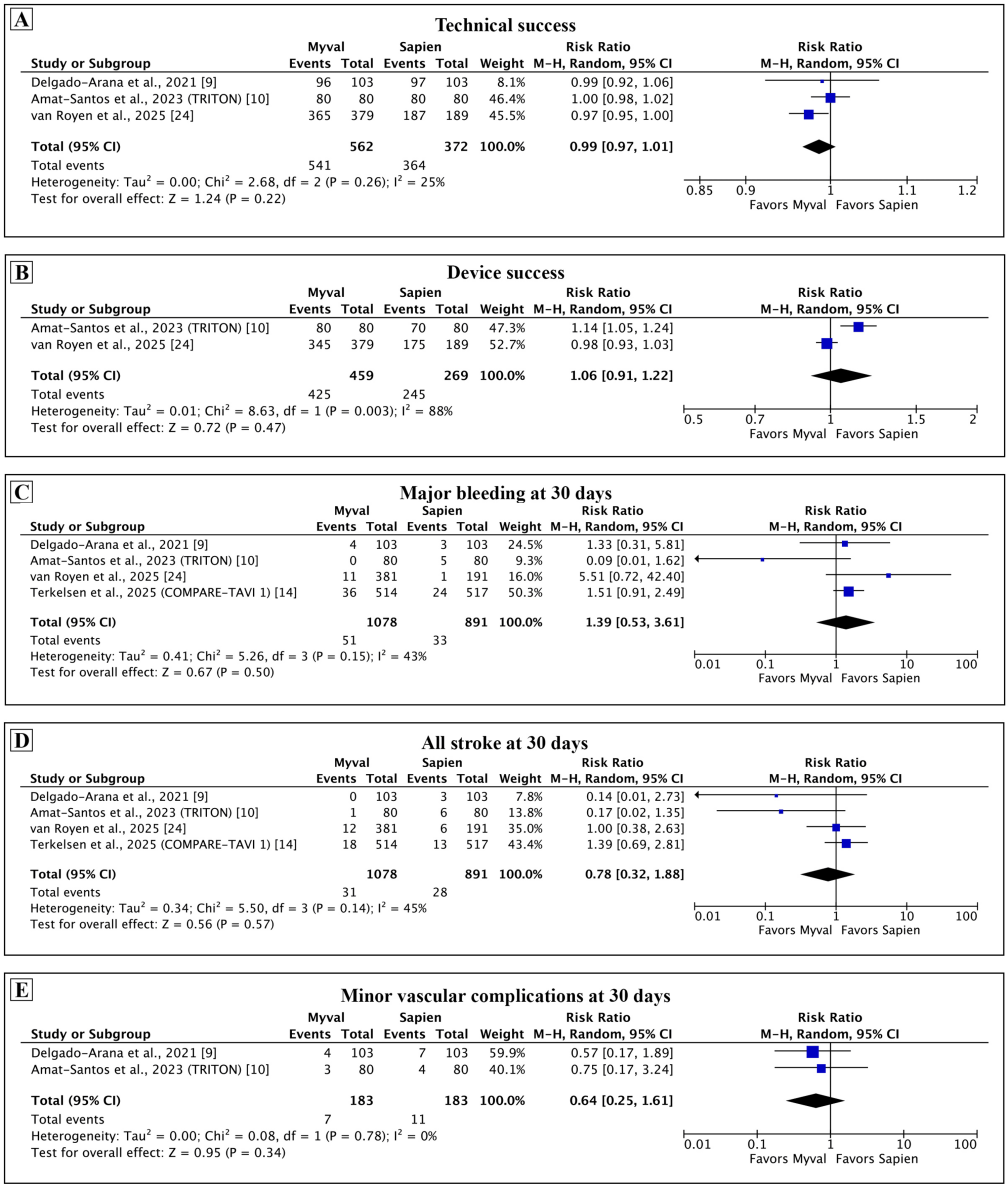
Comparison of secondary outcomes between Myval and Sapien 3 at 30 days. Meta-analysis of the secondary outcomes of technical success (A), device success (B), major bleeding* (C), all stroke (D), and minor vascular complications (E). *Major bleeding in COMPARE TAVI-1 and van Royen et al. was Valve Academic Research Criteria (VARC) 3, 4 bleeding. The TRITON trial reported VARC 2 or higher bleeding. TAVI: transcatheter aortic valve implantation.

**Figure 4:**
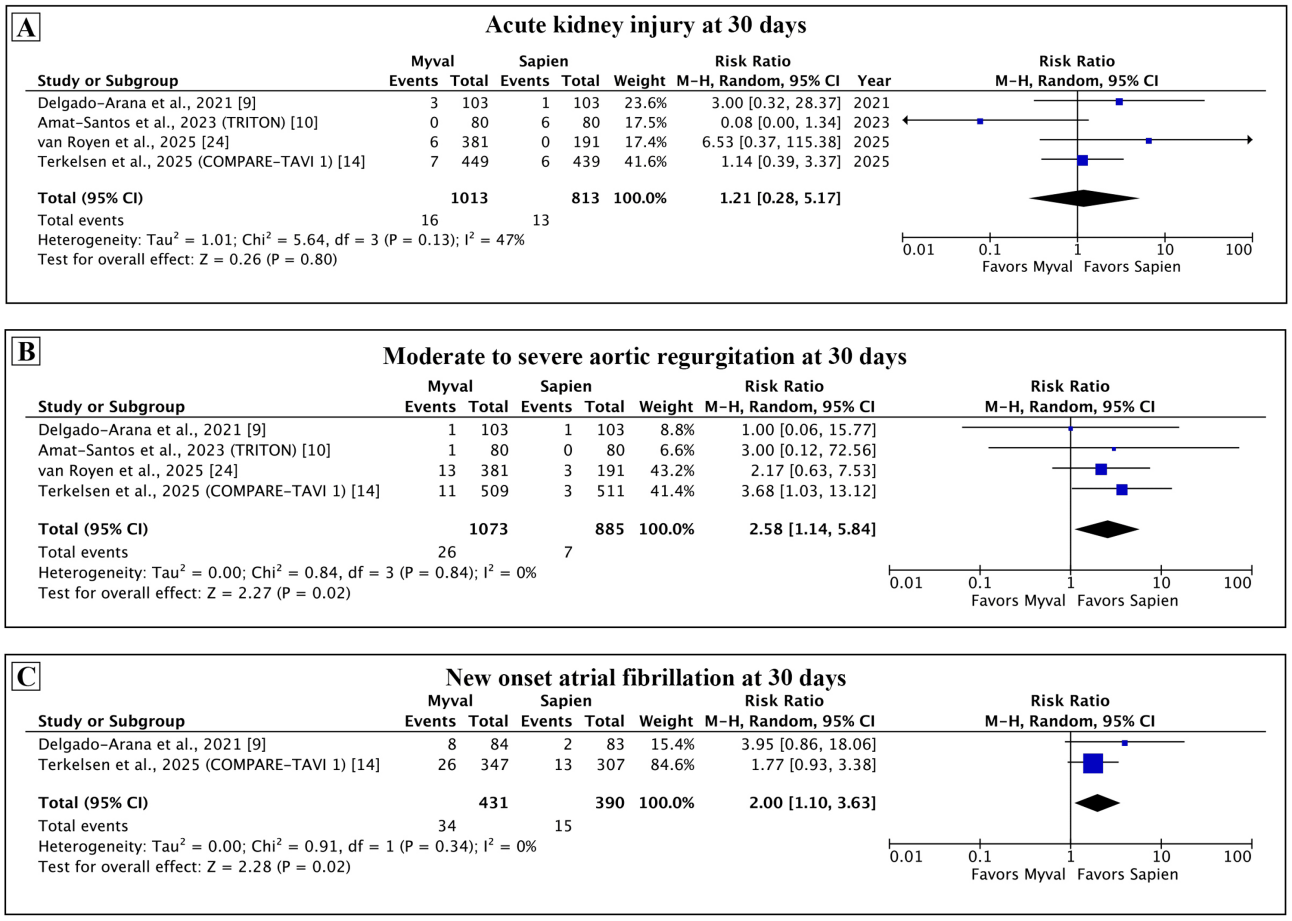
Comparison of secondary outcomes between Myval and Sapien 3 at 30 days. Meta-analysis of the secondary outcomes of acute kidney injury (A), moderate to severe aortic regurgitation (B), and new-onset atrial fibrillation* (C). *The rate of new-onset atrial fibrillation for Delgado-Arana et al. was calculated by excluding the patients with pre-existing atrial fibrillation.

Secondary outcomes: Among the secondary outcomes assessed at 30 days (Figures 3, 4), there was no difference between Myval and Sapien in the technical success (RR: 0.99, 95% CI: 0.97-1.01; p = 0.22; I² = 25%), device success (RR: 1.06, 95% CI: 0.91-1.22; p = 0.47; I² =88%), major bleeding (RR: 1.39, 95% CI: 0.53-3.61; p = 0.50; I² = 43%), all stroke (RR: 0.78, 95% CI: 0.32-1.88; p = 0.57; I² = 45%), minor vascular complications (RR: 0.64, 95% CI: 0.25-1.61; p = 0.34; I² = 0%), and AKI (RR: 1.21, 95% CI: 0.28-5.17; p = 0.80; I² = 47%). Moderate to severe AR (RR: 2.58, 95% CI: 1.14-5.84; p = 0.02; I² = 0%) and new-onset AF (RR: 2.0, 95% CI: 1.10-3.63; p = 0.02; I² = 0%) were more common with Myval as compared with the Sapien. 

**Figure 5:**
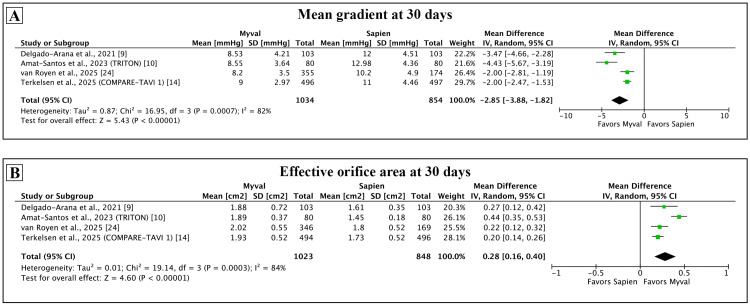
Comparison of hemodynamic outcomes between Myval and Sapien 3 at 30 days. Meta-analysis of the hemodynamic outcomes of mean gradient (A) and effective orifice area (B).

Among the hemodynamic outcomes (Figure 5), the mean gradient was lower (mean difference: -2.85 mmHg, 95% CI: -3.88 to -1.82 mmHg; p < 0.001; I² = 82%) and the EOA was larger with the Myval (mean difference: 0.28 cm^2^, 95% CI: 0.16-0.40 cm^2^; p < 0.001; I² = 84%). These findings were consistent across all individual studies. 

The exploratory analysis of procedural outcomes is presented in the Appendix (see Figure 9). There was no difference in any outcome with similar rates of procedural death (RR: 0.62, 95% CI: 0.12-3.22; p = 0.57; I² = 0%), annular rupture (RR: 0.20, 95% CI: 0.03-1.15; p = 0.07; I² = 0%), coronary occlusion (RR: 0.52, 95% CI: 0.08-3.30; p = 0.49; I² = 18%), valve malposition (RR: 0.62, 95% CI: 0.08-5.06; p = 0.66; I² = 0%) and tamponade (RR: 0.50, 95% CI: 0.19-1.27; p = 0.14; I² = 0%).

Subgroup Analysis and Sensitivity Analysis

**Figure 6:**
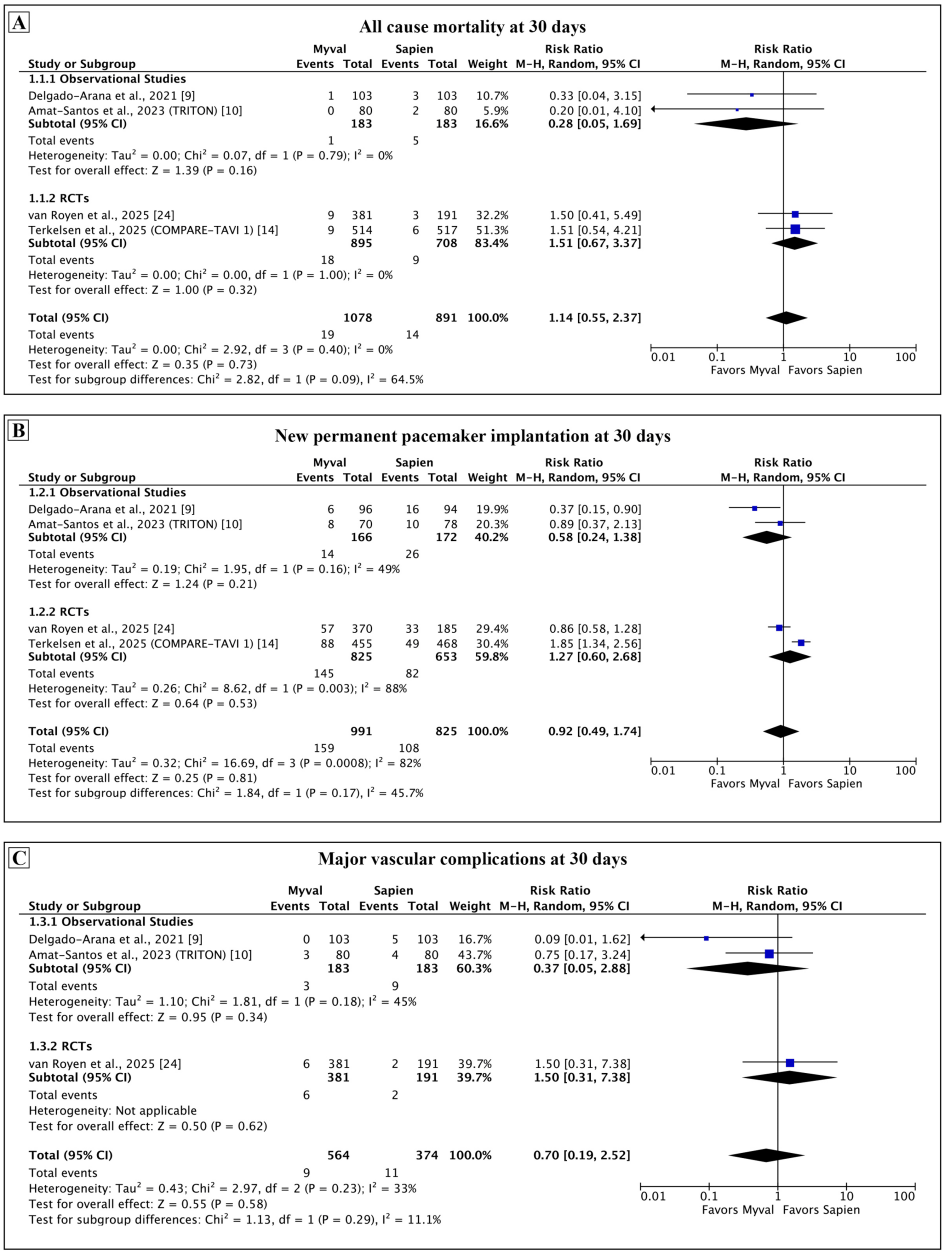
Subgroup analysis for the primary outcomes. Subgroup analysis of the primary outcome based on study type.

**Figure 7:**
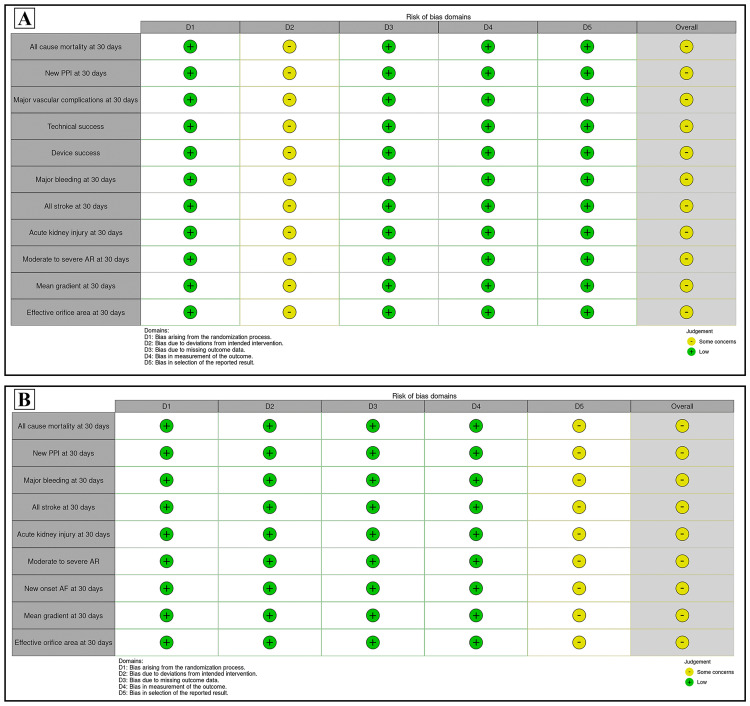
Risk of bias summary for randomized controlled trials. (A)Traffic light plot representing the risk of bias assessment at the outcome level using RoB 2 tool for the LANDMARK study (van Royen et al. 2025) [24]. (B)Traffic light plot representing the risk of bias assessment at the outcome level using RoB 2 tool for the COMPARE-TAVI 1 study (Terkelsen et al. 2025) [14]. AF: atrial fibrillation; AR: aortic regurgitation; PPI: permanent pacemaker implantation; TAVI: transcatheter aortic valve implantation.

**Figure 8:**
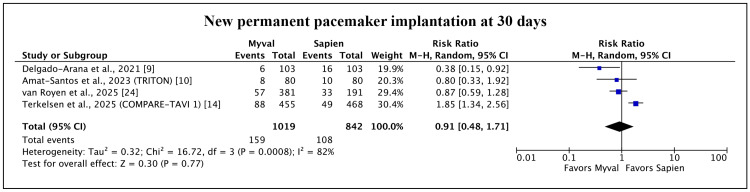
Sensitivity analysis for new permanent pacemaker implantation. Sensitivity analysis for new permanent pacemaker implantation at 30 days using the figures reported by the studies and without excluding patients with pre-existing permanent pacemakers (except for COMPARE-TAVI 1, which reported the rate of new PPI in the primary analysis). PPI: permanent pacemaker implantation.

**Figure 9:**
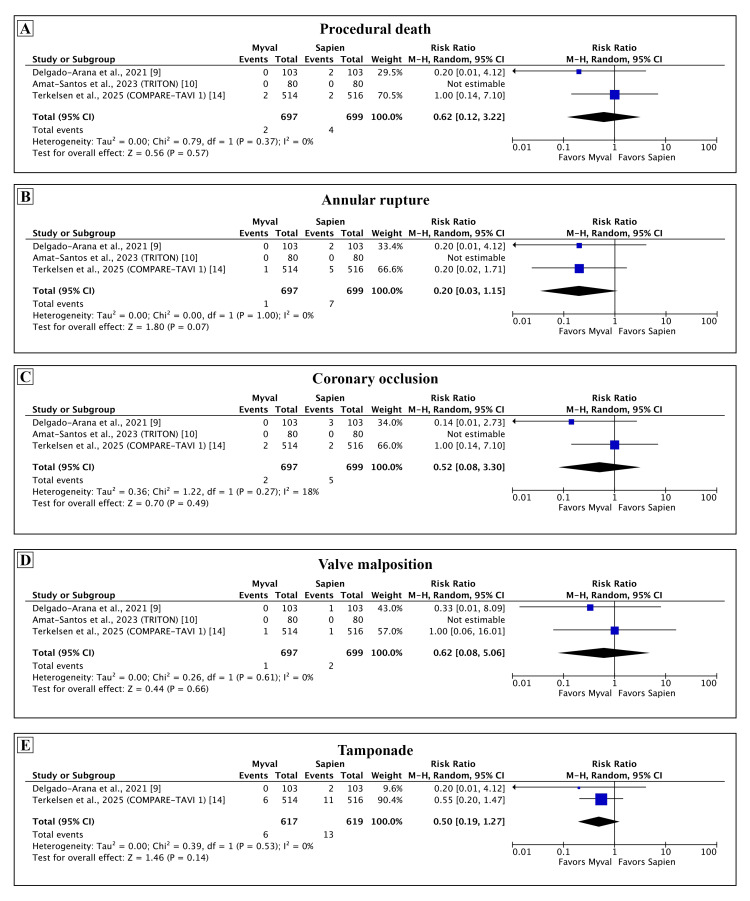
Comparison of procedural outcomes between Myval and Sapien 3. Meta-analysis of the procedural outcomes of procedural death (A), annular rupture (B), coronary occlusion (C), valve malposition (D), and tamponade (E).

**Figure 10:**
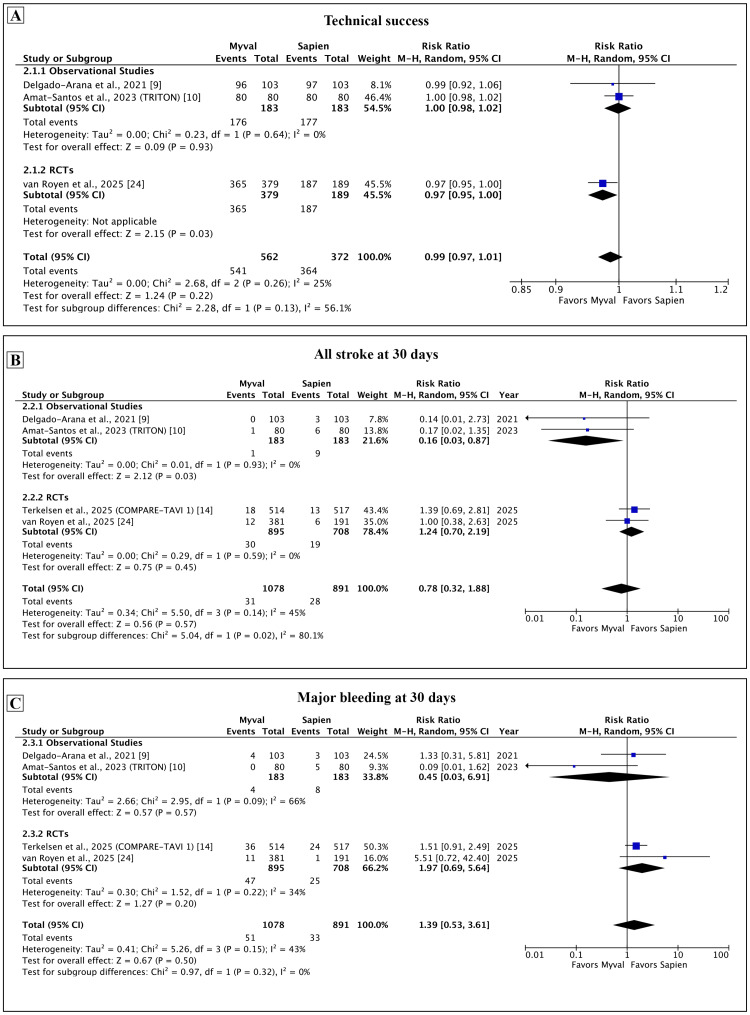
Subgroup analysis of the secondary outcomes. Subgroup analysis of the secondary outcomes at 30 days. (A) Technical success, (B) all stroke, and (C) major bleeding.

**Figure 11:**
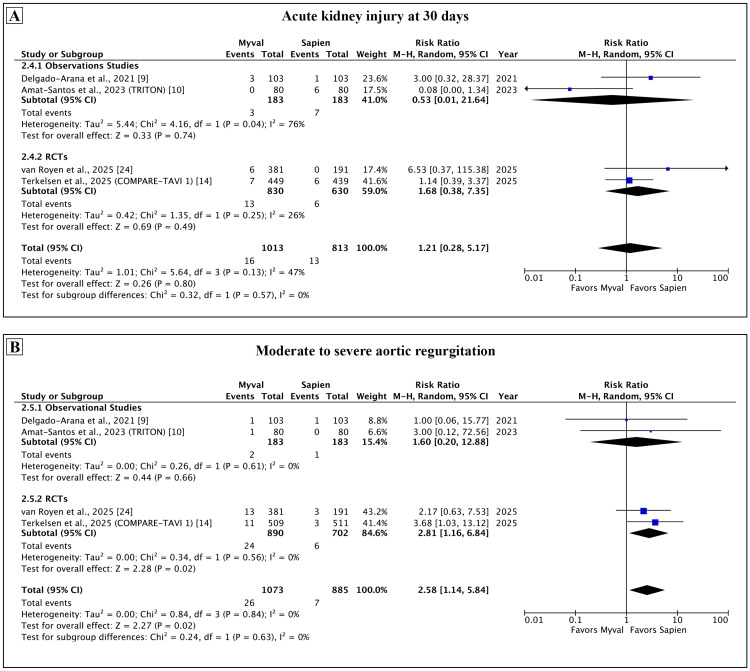
Subgroup analysis for the secondary outcomes. Subgroup analysis of the secondary outcomes of acute kidney injury (A) and moderate to severe aortic regurgitation (B) at 30 days.

**Figure 12:**
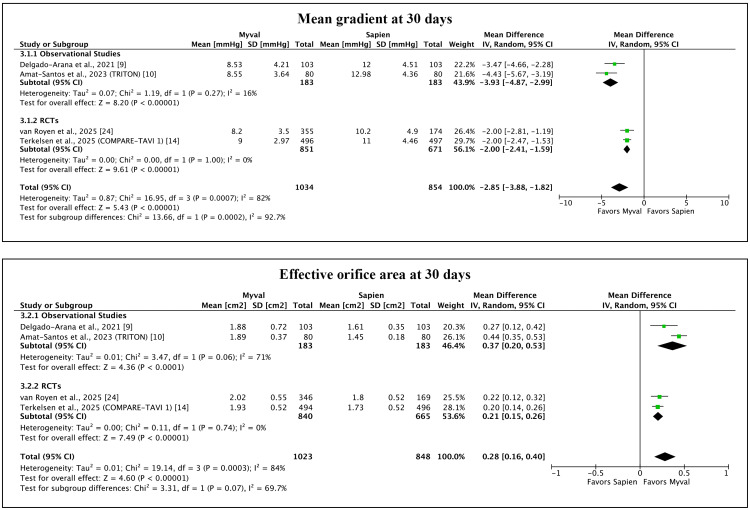
Subgroup analysis for the hemodynamic outcomes. Subgroup analysis for the hemodynamic outcomes of mean gradient (A) and effective orifice area (B) at 30 days.

A predefined subgroup analysis was conducted by grouping RCTs and observational studies separately. The findings of the subgroup analysis for the primary outcomes are presented in Figure 6. There were no significant differences in any primary outcome among the subgroups. The subgroup analysis for the secondary outcomes is presented in the Appendix (see Figures 10-12). We observed inconsistency in outcomes of all strokes at 30 days and moderate to severe AR between the two subgroups, but this difference could be explained by the low event rates and small sample size in the observational studies.

**Figure 13:**
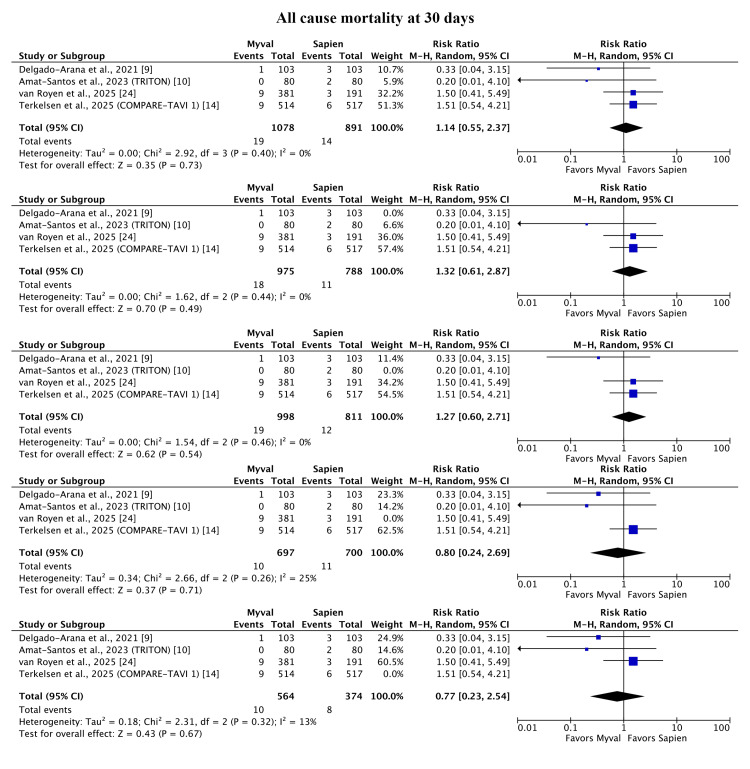
Sensitivity analysis for all-cause mortality at 30 days. Sensitivity analysis of the primary outcome of all-cause mortality at 30 days using the leave-one-out approach.

**Figure 14:**
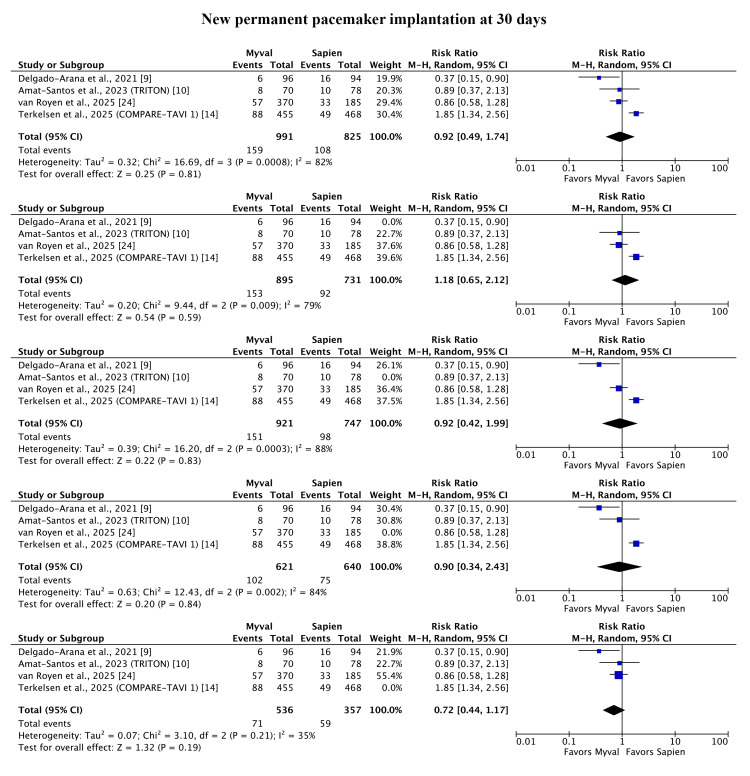
Sensitivity analysis for new permanent pacemaker implantation at 30 days. Sensitivity analysis for the primary outcome of new pacemaker implantation at 30 days using the leave-one-out approach.

**Figure 15:**
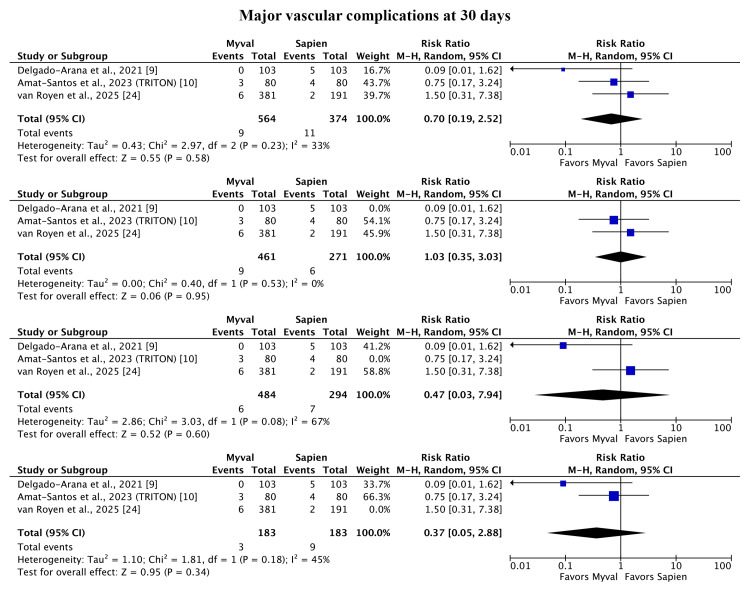
Sensitivity analysis for major vascular complications at 30 days. Sensitivity analysis for the primary outcome of major vascular complications at 30 days using the leave-one-out approach.

A sensitivity analysis for the primary outcomes was performed using the leave-one-out approach (see Appendix, Figures 13-15). The findings remained consistent, suggesting no individual study unduly influenced the results. 

Publication Bias

As only four studies were included in the review, a funnel plot could not be utilized to assess for publication bias [26].

Discussion 

The present systematic review compared the early clinical outcomes between Myval and Sapien 3 THVs. There was no difference in the primary outcomes of all-cause mortality, permanent pacemaker implantation, and major vascular complications assessed at 30 days. Among the secondary outcomes, moderate to severe AR and new-onset AF were more common with Myval. The mean gradient was lower and EOA larger in Myval as compared to Sapien 3. 

Both valves are balloon expandable but with different valve and delivery catheter designs [7]. Myval utilizes a nickel-cobalt alloy frame, whereas the Sapien 3 series features a frame of cobalt-chromium alloy. Furthermore, unlike the Sapien valve, the Myval catheter system does not employ a pusher, and the valve is directly crimped on the balloon. While this permits the undeployed valve to be removed from the body if required, the absence of a pusher may result in less pushability of the device. Hence, there is a trend towards more valve predilation when using the Myval as compared to the Sapien 3. Both valves use proprietary expandable sheaths. Myval's Python sheath has a fixed 14F size for all devices, while the Sapien Sheath comes in 14F and 16F sizes, depending upon the valve size.

Despite differences between the valve systems, the findings of our meta-analysis suggest that important early outcomes between Myval and Sapien 3 are comparable. Moderate to severe AR was more frequent after the Myval, but this appears to be driven by an unusually low rate of AR in the Sapien group (0.8%). The absolute pooled incidence in the Myval group at 2.4% is similar to the previously reported rates of clinically relevant paravalvular leak with Sapien 3 valves [5,27,28]. The rate of new-onset AF in the Myval group was nearly double that of Sapien 3; however, the possible reasons for this finding and its clinical significance remain unclear. 

A notable finding from the present review is the superior hemodynamic performance of the Myval at 30 days. The pooled estimate of the mean difference in EOA was 0.28 cm^2^, with the lower limit of the CI being 0.16 cm^2^. This effect was consistent across all studies. While there is no well-established minimal clinically important difference for EOA, any increment in valve area provides a hemodynamic head start and may be associated with improved valve longevity. Medium and long-term follow-up data with the Myval are limited; however, the COMPARE-TAVI 1 study showed that at one year of follow-up, the Myval group continued to have a lower mean gradient and larger EOA [14]. The superior hemodynamic performance may be explained by the availability of intermediate sizes in the Myval valve size matrix, which were employed in over 40% of the Myval group in three of the four included studies [29]. It remains to be studied, if this hemodynamic benefit observed at the study level and attributable to the use of intermediate Myval sizes, will extend at individual patient level when comparing corresponding sizes of Myval and Sapien 3. 

Of the four studies included in meta-analysis for new PPI at 30 days, all except the COMPARE-TAVI 1 reported the rates of PPI as a proportion of the entire study group. This contrasts with the VARC 3 recommendation, which advises for excluding patients with existing PPIs from the denominator when calculating the rate of new PPIs [8]. We computed the pooled result by excluding patients with existing PPI in the individual studies and found no difference in the rates of PPI at 30 days. 

To our knowledge, the present systematic review is the first one that directly compares two commercially available balloon-expandable valves. Two previous systematic reviews have compared Myval to contemporary valves, including Evolut and Sapien [30,31]. These systematic reviews had broad research questions. Quantitative synthesis was performed by pooling outcomes from both self-expanding and balloon-expanding valves in the control group, leading to significant clinical heterogeneity that dilutes any inference from the pooled estimates [32]. This may explain the contrasting findings in our meta-analysis as compared to the previous ones, which reported lower rates of PPI and higher procedural success with Myval based on a pooled comparison with all other types of contemporary valves. Furthermore, these meta-analyses calculated the PPI rates as a proportion of the total study size, without excluding patients with pre-existing PPIs as required by the VARC-3 definitions [8]. Additionally, the recently published COMPARE-TAVI 1 trial was not included in these systematic reviews.

The strengths of the present review include a robust methodology, prospective protocol registration, and execution in accordance with the PRISMA guidelines. The search strategy was comprehensive, and the inclusion criteria were well-defined. All crucial steps were independently performed by two reviewers. A predefined subgroup analysis was performed, and the ROB 2 tool was used at the outcome level to assess risk of bias.

Limitations of this review include significant methodological, clinical, and statistical heterogeneity among the included studies. There were two observational studies and two RCTs, resulting in methodological heterogeneity. We accounted for this by performing subgroup analysis for the observational studies and RCTs with consistent results for the primary outcome. There was significant clinical heterogeneity among the studies, including differences in the populations studied, operator expertise, and the types of valves used. The TRITON study only included patients with bicuspid aortic valves, while Delgado-Arana et al. excluded patients with bicuspid aortic valves. COMPARE-TAVI was an all-comers study, which also included prosthetic heart valves, while all other studies excluded PHVs. Both first-generation Myval and Myval Octacor were used in LANDMARK and COMPARE TAVI, whereas the other studies utilized the first-generation Myval. The LANDMARK and COMPARE TAVI 1 study also used Sapien 3 Ultra in addition to the Sapien 3 valve. Further, the proportion of intermediate sizes used in the Myval group varied among the studies. All these variables can account for the significant heterogeneity that may not be captured by statistical tests. Nonetheless, despite these factors, we believe a quantitative synthesis was essential to provide pooled estimates of outcomes.

The risk of bias assessment was challenging due to use of different scales for observational studies and RCTs. The observational studies with matched comparison were adjudged to be of high quality according to the NOS; however, a retrospective study design can result in several biases that may not be captured by the NOS [33]. Among the RCTs, both had some concerns with regards to the risk of bias. The LANDMARK trial had some concerns due to deviation from the intended intervention, while the COMPARE-TAVI 1 study had some concerns arising out of risk of bias in the selection of reported results [14,24].

Our study carries implications for future research. A medium and long-term follow-up of patients undergoing TAVI with Myval is necessary. A recent retrospective analysis demonstrated that the latest iteration of the Sapien 3 Ultra Resilia valve has similar hemodynamic outcomes compared to the Myval [34]. Likewise, the second-generation Myval Octacor also has certain advantages, including larger skirt size, decreased foreshortening, and the option for commissural alignment, which may theoretically improve hemodynamic performance over the long term [35,36]. Hence, a direct comparison between Myval Octacor and Sapien 3 Ultra Resilia may be relevant to inform future practice. As both THV systems have significant design differences and, therefore, likely costs, a cost-effectiveness analysis of Myval versus Sapien 3 is also warranted. 

## Conclusions

To conclude, in this systematic review and meta-analysis, we report early outcomes with Myval compared to the Sapien 3 valve for TAVI in severe AS. There was no difference in the primary clinical outcomes at 30 days, with comparable rates of all-cause mortality, new PPI, and major vascular complications between the two valve systems. Moderate to severe AR and new-onset AF were lower with Sapien 3. Myval had a better hemodynamic performance with a lower mean gradient and higher effective orifice area at 30 days.

## Appendices

**Table 2:** Search strategy for various databases.

Database	Search string
Pubmed	"Aortic Valve Stenosis" [Mesh] OR "Aortic Valve Disease" [Mesh] OR "Aortic stenosis" OR "AS" OR "AV stenosis" AND "Transcatheter Aortic Valve Replacement"[Mesh] OR "transcatheter aortic valve implantation" OR "transcatheter aortic valve replacement" OR "TAVI" OR "TAVR" OR "THV" OR ("transcatheter" AND "aortic" AND "valve" and "implantation") OR ("transcatheter" AND "aortic" AND "valve" AND "replacement") AND "MyVal" OR "Myval" OR "Meril valve" OR "Meril" OR "Meril THV" OR "Myval THV"
Embase	'aortic valve stenosis'/syn OR ‘Aortic stenosis’ OR ‘AS’ OR ‘AV stenosis’ OR (‘aortic’ AND ‘stenosis’) AND 'transcatheter aortic valve implantation'/syn OR ‘transcatheter aortic valve implantation’ OR ‘transcatheter aortic valve replacement’ OR ‘TAVI’ OR ‘TAVR’ OR ‘THV’ OR (‘transcatheter’ AND ‘aortic’ AND ‘valve’ and ‘implantation’) OR (‘transcatheter’ AND ‘aortic’ AND ‘valve’ AND ‘replacement’) AND ‘MyVal’ OR ‘Myval’ OR ‘Meril valve’ OR ‘Meril’ OR ‘Meril THV’ OR ‘Myval THV’
Cochrane Library	MeSH descriptor: [Aortic Valve Stenosis] explode all trees OR (“Aortic stenosis” OR “AS” OR “AV stenosis”):ti,ab,kw AND MeSH descriptor: [Transcatheter Aortic Valve Replacement] explode OR (“transcatheter aortic valve implantation” OR “transcatheter aortic valve replacement” OR “TAVI” OR “TAVR” OR “THV” OR (“transcatheter” AND “aortic” AND “valve” and “implantation”) OR (“transcatheter” AND “aortic” AND “valve” AND “replacement”)):ti,ab,kw AND (“MyVal” OR “Myval” OR “Meril valve” OR “Meril” OR “Meril THV” OR “Myval THV”):ti,ab,kw

**Table 3:** List of relevant excluded reports.

Study	Authors	Reason for exclusion
Myval versus alternative balloon- and self-expandable transcatheter heart valves: A central core lab analysis of conduction disturbances [11]	Santos-Martinez et al. (2022)	The study focused on the rate of permanent pacemaker implantation. The study reported in-hospital outcomes and did not report 30-day outcomes.
German experience with a novel balloon-expandable heart valve prosthesis for transcatheter aortic valve implantation-outcomes of the MYLAND (MYvaL germAN stuDy) Study [12]	Ubben et al. (2024)	The study reported in-hospital outcomes and did not report 30-day outcomes.
LANDMARK comparison of early outcomes of newer-generation Myval transcatheter heart valve series with contemporary valves (Sapien and Evolut) in real-world individuals with severe symptomatic native aortic stenosis: a randomised non-inferiority trial [13]	Baumbach et al. (2024)	Primary report for the LANDMARK study, but did not provide a direct comparison between Myval and Sapien 3, which was reported by van Royen et al. and included in the systematic review.
Quantitative angiographic assessment of aortic regurgitation after transcatheter aortic valve implantation among three balloon-expandable valves [37]	Kawashima et al. (2021)	The study reported rates of acute aortic regurgitation after transcatheter aortic valve implantation based on videodensitometric assessment of the catheter aortogram. The study did not report 30-day outcomes.
Need for long-term permanent pacemaker and its association with mortality in patients undergoing transcatheter aortic valve implantation [38]	Ozbek et al. (2023)	The study provided long-term rates of PPI between different transcatheter heart valves. The study did not report 30-day outcomes.
Single center experience of transcatheter aortic valve implantation in severe aortic stenosis (2009–2021) [39]	Tey et al. (2022)	The study did not report comparison between Myval and Sapien 3.
One-year outcomes for patients undergoing transcatheter aortic valve replacement: the Gulf TAVR Registry [40]	Alasnag et al. (2022)	The study did not report comparison between Myval and Sapien 3.
Win ratio analysis of the LANDMARK trial [41]	Tobe et al. (2025)	Substudy of the landmark trial.

**Table 4:** Additional study characteristics. TAVI: transcatheter aortic valve implantation; VARC: Valve Academic Research Consortium; AR: aortic regurgitation; THV: transcatheter heart valve; AS: aortic stenosis.

Study and author	Inclusion criteria	Exclusion criteria	Primary endpoint	Secondary endpoint
Delgado-Arana et al. 2021 [9]	Consecutive real-world symptomatic patients with severe aortic stenosis of the native valve who received balloon-expandable TAVR (nickel-cobalt frame or chromium-cobalt frame) devices across nine European centers using both device	Bicuspid aortic valve	The primary endpoint was to compare 30-day degree of AR, residual transvalvular gradient, estimated aortic valve area and prosthesis-patient mismatch rate between Myval and Sapien 3 after matching for baseline anatomical and functional differences	Periprocedural and 30-day outcomes according to VARC-2 criteria
Amat-Santos et al. 2023 (TRITON) [10]	Real-world consecutive symptomatic patients with severe aortic stenosis and BAV morphology were recruited	No obvious exclusion criteria, as the study was an all-comers study with patients of bicuspid aortic valve stenosis	The primary endpoint was the composite endpoint of 30-day device success as defined by the VARC-3 criteria	Secondary endpoints were the composite and individual components of early safety at 30 days; hemodynamic performance at 30-days as assessed by transthoracic echocardiography
van Royen et al. 2025 (LANDMARK) [24]	Adults with severe symptomatic native AS were if eligible for TAVI with Myval, Sapien 3 and Evolut platforms	Any condition, which in the investigator's opinion, would preclude safe participation of patient in the study	The primary combined safety and effectiveness endpoint at 30 days was a composite of all-cause mortality, all stroke, bleeding (VARC types 3 and 4), acute kidney injury (stages 2, 3, and 4), major vascular complications, moderate or severe prosthetic valve regurgitation (PVR), and conduction system disturbances resulting in a new permanent pacemaker implantation (PPI) as per VARC-3	Secondary endpoint included components of the primary endpoint, technical success, device success and early safely endpoints at 30-day follow-up
Terkelsen et al. 2025 (COMPARE-TAVI 1) [14]	Adult patients scheduled for transfemoral TAVI and eligible for either Sapien 3 or Myval	No obvious exclusion criteria, as the study was an all-comers study	The primary endpoint was a composite of all-cause death, stroke, moderate or severe aortic regurgitation, or moderate or severe haemodynamic THV deterioration at one year according to the VARC-3 criteria	Secondary endpoints were the proportion of patients with successful implantation of the chosen valve, pacemaker implantation and TAVI-related complications

**Table 5:** Baseline clinical characteristics in included studies. MYL: Myval; SAP: Sapien 3; PPI: permanent pacemaker implantation; COPD: chronic obstructive pulmonary disease; TIA: transient ischemic attack; CAD: coronary artery disease; AF: atrial fibrillation; NYHA: New York Heart Association; STS: Society of Thoracic Surgeons.

Study and author	Patients (n)		Mean age (years)		Males (%)		Females (%)		Existing PPI (%)		COPD (%)		Previous stroke/TIA (%)		CAD (%)		Prior AF (%)		NYHA III or IV (%)		Euroscore II (%)		STS score (%)	
	MYL	SAP	MYL	SAP	MYL	SAP	MYL	SAP	MYL	SAP	MYL	SAP	MYL	SAP	MYL	SAP	MYL	SAP	MYL	SAP	MYL	SAP	MYL	SAP
Delgado-Arana et al. 2021 [9]	103	103	81 ± 6.5	80.6 ± 6.2	56.3	63.1	43.7	36.9	6.8	8.7	10.7	14.6	13.6	21.4	41.7	36.9	27.2	26.2	47.6	44.7	NA	NA	3.3 (2.2-5.4)	3.5 (2.1-4.8)
Amat-Santos et al. 2023 (TRITON) [10]	80	80	77.04 ± 5.66	77.61 ± 6.28	72.5	76.3	27.5	23.8	12.5	2.5	12.7	15.2	3.8	3.8	41.3	45	22.5	21.3	42.5	48.8	4.38 ± 4.80	2.94 ± 2.34	3.67 ± 2.26	2.20 ± 0.86
van Royen et al. 2025 (LANDMARK) [24]	384	192	80.0 ± 5.7	81.1 ± 5.4	49.7	55.2	50.3	44.8	2.9	3.1	10.9	10.4	3.4	1.6	NA	NA	24.5	23.4	NA	NA	NA	NA	2.6 (1.7-4.0)	2.6 (1.8-4.0)
Terkelsen et al. 2025 (COMPARE-TAVI 1) [14]	514	517	81.9 (77.8-85.2)	81.1 (77.5 - 84.8)	60	60	40	40	13.4	10.4	NA	NA	NA	NA	18.8	19.6	32.5	40.6	38.9	40.8	2.5 (1.5-4.3)	2.6 (1.7-4.3)	2.3 (1.6-3.8)	2.4 (1.6-3.5)

**Table 6:** Baseline echocardiographic characteristics. LVEF: left ventricular ejection fraction.

Study and author	Patients (n)		LVEF (%)		Peak gradient (mmHg)		Mean gradient (mmHg)		Aortic valve area (mm^2^)	
	Myval	Sapien	Myval	Sapien	Myval	Sapien	Myval	Sapien	Myval	Sapien
Delgado-Arana et al. 2021 [9]	103	103	55.5 ± 10	55.5 ± 12.9	67 (55-78)	71 (61-79)	40 (34-49)	43 (36-50)	0.7 (0.57-0.88)	0.7 (0.6-0.8)
Amat-Santos et al. 2023 (TRITON) [10]	80	80	56.29 ± 11.86	59.44 ± 10.24	80.35 ± 19.57	78.76 ± 24.30	52.33 ± 12.72	51.84 ± 14.56	0.66 ± 0.16	0.66 ± 0.17
van Royen et al. 2025 (LANDMARK) [24]	384	192	NA	NA	NA	NA	39.9 ± 14.0 (n = 368)	39.2 ± 14.2 (n = 184)	0.74 ± 0.22 (n = 364)	0.70 ± 0.21 (n = 180)
Terkelsen et al. 2025 (COMPARE-TAVI 1) [14]	514	517	58 (47-66); n = 514	56 (47-63); n = 515	72 (59-87); n = 510	73 (59-88); n = 516	47 (38–57); n = 509	46 (37–56); n = 516	0·7 (0∙6–0∙9); n = 506	0∙7 (0∙6–0∙8); n = 514

**Table 7:** Baseline CT characteristics. AU: Agatston units; CT: computed tomography.

Study and author	Patients		Bicuspid valves (%)		Valve-in-valve (%)		Aortic annulus area (mm^2^)		Small annuli (area < 430 mm^2^) (%)		Average diameter (mm)		Annulus perimeter (mm)		Aortic valve calcium score (AU)	
	Myval	Sapien	Myval	Sapien	Myval	Sapien	Myval	Sapien	Myval	Sapien	Myval	Sapien	Myval	Sapien	Myval	Sapien
Delgado-Arana et al. 2021 [9]	103	103	0	0	0	0	467 (384-516)	456 (399-511)	NA	NA	24.4 ± 2.6	24.6 ± 2.7	NA	NA	2132 (688-2480)	2001 (1269-3077)
Amat-Santos et al. 2023 (TRITON) [10]	80	80	100	100	0	0	540 ± 112	531 ± 114	NA	NA	NA	NA	82.26 ± 8.90	81.31 ± 8.54	4936 ± 2611	5011 ± 2442
van Royen et al. 2025 (LANDMARK) [24]	384	192	6.0	7.3	0	0	470.5 ± 80.0	469.3 ± 82.6	32.6	33.3	NA	NA	77.8 ± 6.7	77.7 ± 6.9	NA	NA
Terkelsen et al. 2025 (COMPARE-TAVI 1) [14]	514	517	9.3	9.7	3.9	3.9	472 (415–531); n = 494	485 (415–550); n = 497	30.6	31.2	24·9 (23∙1–26∙3); n = 492	25·0 (23∙1–26∙6); n = 494	79 (74–83); n = 493	80 (74–85); n = 497	NA	NA

**Table 8:** Procedural characteristics. *Based on as-treated analysis (n = 507) and includes extra-large sizes of Myval, which were used in 10 patients.

Study and author	Patients		Transfemoral access (%)		Predilation (%)		Postdilation		Intermediate sizes
	Myval	Sapien	Myval	Sapien	Myval	Sapien	Myval	Sapien	Myval
Delgado-Arana et al. 2021 [9]	103	103	100	100	36.9	24.3	6.8	5.8	44.3
Amat-Santos et al. 2023 (TRITON) [10]	80	80	100	100	70.1	44.2	15.4	12.8	25.2
van Royen et al. 2025 (LANDMARK) [24]	384	192	99.7	97.5	43.3	30.7	10.0	10.1	48
Terkelsen et al. 2025 (COMPARE-TAVI 1) [14]	514	516	100.0	100.0	44.7	20.7	13.2	11.4	41.8*

**Table 9:** Risk of bias summary for the observational studies. Risk of bias assessment for the observational studies as assessed using the Newcastle-Ottawa Scale.

Study	Selection				Comparability	Outcome			Total score	Quality of the study
	Representativeness of the exposed cohort	Selection of the non-exposed cohort	Ascertainment of exposure	Demonstration that outcome of interest was not preset at start of study	Comparability of cohorts on the basis of the design or analysis	Assessment of outcome	Was follow-up long enough for outcomes to occur	Adequacy of follow up of cohorts		
Delgado-Arana et al. 2021 [9]	1	1	1	1	2	1	1	1	9	High
Amat-Santos et al. 2023 (TRITON) [10]	0	1	1	1	2	1	1	1	8	High

Subgroup analysis for the secondary outcome
